# Supplementary material for: Digital economy and green total factor productivity in China
Source: PLoS One. 2024 Mar 1;19(3):e0299716. doi: 10.1371/journal.pone.0299716 (PMC10906909; doi:10.1371/journal.pone.0299716)
Supplement: Attachment — Submitted filename: Response to Reviewers.docx [file pone.0299716.s002.docx]

Response to Reviewers

We sincerely thank the editor and the reviewer for their valuable suggestions that we have used to improve the quality of our manuscript.The reviewer comments are laid out below in italicized font and specific concerns have been numbered.Our response is given in normal font.In this revised version, changes to our manuscript are all highlighted within the document by using blue-colored text. Point by point responses to the nice editor and the nice reviewer are listed below this letter.

***Review Comments to the Author***

*This paper studies the relationship between the Digital Economy and Green Total Factor Productivity to promote high-quality regional development. The context of the research is novel, and it is very interesting. Furthermore, it has great motivation for the development of society.*

**Response**

We thank you for your appreciation of the study and we have seriously considered your primary concerns with our manuscript.According to the reviewers’comments, we have made extensive modifications.And we have defined the research framework and supplemented extra variables to make our results convincing.

*1. Is there a basis for the measurement of the digital economy and how is the selection of indicators made? What are the similarities and differences with the indicator designs currently considered in other studies? How do the results of the digital economy evaluation reflect consistency with reality?*

**Response**

We appreciate the reviewer’s insightful comments.The reviewers' comments indicate that our exposition needs further fleshing out.We adopt the professional comments of the reviewer and provide the following explanation.Based on the meaning of digital economy, the Internet is an important carrier for the development of digital economy.The construction of digital economy indicators at the inter-provincial level takes Internet informatization as the core of the measurement.For example, Liu(2020)published an article on constructing the digital economy index through informatization and digitization indicators.For the selection of digital economy indicators, in line with the literature (Chen, 2022; Liu et al., 2020), our indicators include both total telecommunication services per capita, the share of the number of people in information computer services, and other information technology indicators.Informationization indicators reflect the current degree of informationization in the region and reflect the basic support environment of the development of the digital economy.Our indicators also include the number of Internet broadband access users, the number of cell phone users and other Internet development indicators, which fully consider the infrastructure of the digital economy and reflect the conditions for the development of the digital economy.

*2. The process of data analysis is a bit tedious. With only six variables, is it necessary to use the VIF test to test for multicollinearity? In addition, GMM estimation was chosen because of the significance of the coefficients? Is the estimated model calculated by GMM method significant? Please analyze the reasons for choosing GMM estimation and explain the advantages of GMM compared to other methods.*

**Response**

We appreciate the reviewer’s professional comments.we have followed the reviewer’suggestion by adding three control variables in all the models of the revised manuscript on the basis of prior literature (Line 419-426 of the revised manuscript), as below:(1) Foreign direct investment (for), foreign investment in regional green innovation affects the improvement of green total factor productivity.(2) the level of science and technology (tech), the higher the level of science and technology, the stronger the green technology innovation capacity, thus affecting the green total factor productivity.(3) The level of opening up (open), the higher degree of openness between the regions of the faster flow of factors of production, conducive to enhance green total factor productivity.In addition to the three control variables mentioned above, we used sample exclusion, model replacement, and time reduction for robustness testing.The results in columns (15)-(17) of Table 10 in the revised manuscript indicate that the results of our study are robust after adding control variables.

Thank you to the reviewer for your professional comments.We also note the necessity for multicollinearity tests and the issue of significance of GMM method.With your prompting, we make the following responses and modifications: (1) Regarding the necessity of VIF test. We include the VIF test in the article to mitigate the effect of multicollinearity.We have added some explanations for the VIF test results in the revised manuscript.(2) On the model selection and significance of GMM.The results of the estimated models we calculated using the GMM method are all significant.We chose the GMM method estimation not because of the significance of the coefficients.We choose GMM estimation for the following reasons: first, green total factor productivity incorporates resource and environmental factors into the analysis framework.Changes in resources and environmental factors are a dynamic process.The change in green total factor productivity in the previous period affects green total factor productivity in the current period.Therefore, to reflect the dynamic characteristics of green total factor productivity over time, we use the GMM method for estimation.Second, the explanatory variables contain the lagged terms of the explained variables.This can cause the explanatory variables to be correlated with the stochastic perturbation terms, which can lead to endogeneity problems.In this case, we use methods such as ordinary least square and maximum likelihood estimate leading to biased estimation results.The GMM approach overcomes the endogeneity problem in the regression process by using appropriate instrumental variables.Therefore, we use the GMM method.

we have followed the reviewer’s suggestion by adding vif test of the revised manuscript on the basis of prior manuscript (Line 457-465 of the revised manuscript), as below: “VIF tests of the main variables are provided in Table 4, intending to preliminarily check for the presence of multicollinearity. To verify there is no multicollinearity, the VIF test was conducted in this study. All variables have VIF < 5, and the mean VIF is 1.980, indicating that multicollinearity is not a problem. ”

*3. Please explain the philosophy of the robustness test. Are the three robustness analyses here intended to illustrate the robustness of the method or the robustness of the results? I don't quite understand the meaning of including this test here for the full analysis.*

**Response**

The reviewer' comments give us important insights.The purpose of the robustness test in this paper needs further explanation.Robustness tests verify the reliability of the findings through a series of methods.Three robustness analyses are performed to illustrate the robustness of the results.Consistent with the literature (Han, 2021; LYu et al., 2022), in the first robustness analysis, we take the exclusion of special samples to verify the robustness of the results because Beijing, Shanghai and Guangdong have higher levels of digital economy than other provinces. In the second robustness analysis, to avoid significant results due to the choice of a specific model, we take a substitution model to verify the robustness of the results.In the third robustness analysis, we take a shortened time to verify the robustness of the results in order to avoid chance results due to the selection of a specific sample period.

*4. In addition, the second part of this paper has garbled codes and the formula expressions have disappeared.*

**Response**

We are very grateful to the reviewer for careful review.We have re-reviewed the submitted manuscripts.We found that the problem you pointed out due to the software version (file conversion).We apologize to you for this oversight.We have also done several checks elsewhere in the text to ensure that such problems do not occur again.

*5. Please check the focus of the article and sort out the analysis process of the article.*

**Response**

We thank the reviewer for your professional comments.The logical framework of the original manuscript need to be further clarified and the focus of the study sharpened.Based on the reviewer' comments, we have revised the introduction of the article (lines 41-128 of the revised version).We also adjusted the logical framework of the article.The details are as follows: The theoretical introduction is stated in Section 1.The introduction focuses on the research background of the digital economy and green total factor productivity.Based on this, we clarify the research methodology and address the relevant questions.Section 2 presents the literature review and research hypotheses.We combed through the literature on the digital economy and green total factor productivity to discover the innovative points of the study, and then proposed the research hypothesis.Section 3 describes the data sources and model construction.We construct indicators of the digital economy and green total factor productivity based on the data.We select the variables and thus construct dynamic panel models and threshold models.Section 4 analyzes the empirical results.We describe the impact of the digital economy on green total factor productivity and explore the role of the digital economy on green total factor productivity in terms of the innovation environment and institutional environment.We perform robustness tests to ensure the robustness of the results.Section 5 provides a discussion of further analysis. We use quantile regression to distinguish whether there is a difference in the impact of the digital economy on green total factor productivity when it varies.Finally, the conclusions and proposals for further studies stand in Section 6.We summarize the results of the previous study on the digital economy and green total factor productivity.Then, we make the corresponding recommendations.

Again, we thank you for all the above constructive comments.In addition, during the revision process, we added the necessary theoretical literature.We also checked the full text of words and phrases to further improve the standardization and readability of the paper.If you find any problems in the follow-up review, please let us know in time.We will certainly continue to revise and improve the paper according to your requirements in order to make it meet the publication standards of the Plos One journal.
