# Supplementary material for: Digital economy and green total factor productivity in China
Source: PLoS One. 2024 Mar 1;19(3):e0299716. doi: 10.1371/journal.pone.0299716 (PMC10906909; doi:10.1371/journal.pone.0299716)
Supplement: Attachment — Submitted filename: Responses to Reviewers.docx [file pone.0299716.s003.docx]

**Responses to Reviewers**

We sincerely thank the editor and the reviewers for their valuable suggestions that we have used to improve the quality of our manuscript. The reviewers’ comments are laid out below in italicized font and specific concerns have been numbered. Our response is given in normal font. In this revised version, changes for reviewer #1’s comments to our manuscript are highlighted within the document by using red-colored text. Changes for reviewer #2’s comments to our manuscript are highlighted within the document by using blue-colored text. Point by point responses to the editor and the reviewers are listed below this letter.

***Reviewer*** ***#1’s Comments to the Author***

*The overall content has been updated and improved. After carefully checking the related writing details and formulas, it can be accepted. Additionally, concerning issues related to publication, research ethics, or publication ethics, the author should pay attention. If there are similar studies related to these topics, please include them in the list of references.*

**Response**

We thank you for your appreciation of the study and we have seriously considered your primary concerns with our manuscript. According to the reviewer #1’s comments, we have made extensive modifications. We pay more attention to issues related to publication, research ethics, or publication ethics. And we have tried to find similar studies related to these topics. We have supplemented these studies to the list of references. The references are as follows:

Lyu Y, Wang W, Wu Y, Zhang J. How does digital economy affect green total factor productivity? Evidence from China. Science of The Total Environment. 2023; 857: 159428.

Zhang C, Wang L, Yu S, Zhang Y. Energy production factors and total factor productivity in China: Evidence from a new perspective. Energy Economics. 2019; 78: 42–52.

Xiaoqi L, Enshuo L. Spatial Effects of digital economy and foreign direct investment—A u-shaped relationship. Information Systems and Economics. 2023; 4: 30–38.

Xu J, Li W. Study on the impact of digital economy on innovation output based on dynamic panel data model. European Journal of Innovation Management. 2023.

***Reviewer*** ***#2’s Comments to the Author***

*1. Digital economy plays an important role in improving regional green total factor productivity and promoting high-quality economic development. Do not claim the outcome at the start of the sentence.*

**Response**

We appreciate the reviewer#2’s insightful comments. We shouldn't claim the outcome at the start of the sentence. Therefore, we have followed the reviewer#2’s suggestion by revising the sentence of the revised manuscript (Line 9-11 of the revised manuscript), as below: “The development of information technology has created conducive conditions for the digital economy. The digital economy is regarded as a critical pathway for transforming traditional economic models.”

*2. Why this study is important: mention the gap before the objective.*

**Response**

We adopt the reviewers' suggestions, and add the importance of this study and the gap before the objective in the revised manuscript (Line 11-15 of the revised manuscript), as below: “Green total factor productivity serves as an indicator for assessing the quality of economic development. During pivotal periods of economic transition, the digital economy and green total factor productivity have emerged as two prominent themes for achieving sustainable economic development. But the impact of digital economy on green total factor productivity is less discussed.”

*3. Do not use abbreviations in the abstract like the SBM-DDF model.*

**Response**

We are very grateful to the reviewer#2 for careful review. We have re-reviewed the submitted manuscripts. We found that the problem you pointed out due to our carelessness in the format of writing. We apologize to you for this oversight. We have also done several checks elsewhere in the text to ensure that such problems do not occur again. The full name of SBM-DDF model is the Slack based measure-Directional distance function model. (Line 21-22 of the revised manuscript)

*4. According to a further research Which future research can authors explain?*

**Response**

We have followed the reviewer’s suggestion by adding a lot of explanations about the future research in the revised manuscript (Line 23-29 of the revised manuscript), as below: “Generalized Method of Moments model is constructed to carry out an empirical study on the impact of digital economy on green total factor productivity. This paper constructs a panel threshold model with innovation environment and institutional environment as threshold variables. In further analysis, this paper employs panel quantile regression for the empirical analysis of the impact of the digital economy on green total factor productivity. Further analysis elucidates the evident disparities in the influence of the digital economy on green total factor productivity at various levels.”

*5. What is this institutional environment?*

**Response**

We appreciate the reviewer#2’s insightful comments. We have followed the suggestion. We explain the definition of institutional environment in the abstract. We add the sentence of the revised manuscript (Line 15-16 of the revised manuscript), as below: “The institutional environment encompasses the aggregate of economic, political, social, and legal rules.”

*6. What’s the innovation environment?*

**Response**

We appreciate the reviewer#2’s insightful comments. We have followed the suggestion. We explain the definition of innovation environment in the abstract. We add the sentence of the revised manuscript (Line 17-18 of the revised manuscript), as below: “Innovation environment refers to a confluence of conditions shaped by factors such as talent, funding, cultural atmosphere and government policies, all of which collectively support innovative activities within a region.”

**Introduction**

*7. Examples of such mechanisms include the relationship between the digital economy and technological innovation, financial infrastructure, environmental regulations, foreign direct investment, and more. It is important to add the reference for the above statement.*

**Response**

We have followed the reviewer#2’s suggestion by adding the references about the relationship between the digital economy and technological innovation, financial infrastructure, environmental regulations, foreign direct investment. The references are as follows:

Li J, Chen L, Chen Y, He J. Digital economy, technological innovation, and green economic efficiency—Empirical evidence from 277 cities in China. MDE Manage Decis Econ. 2022;43: 616–629. doi:10.1002/mde.3406

Veselovsky MY, Pogodina TV, Ilyukhina RV, Sigunova TA, Kuzovleva NF. Financial and economic mechanisms of promoting innovative activity in the context of the digital economy formation. J Entrep. Sustain Issu. 2018;5: 672–681. doi:10.9770/jesi.2018.5.3(19)

Wang J, Zhang G. Can environmental regulation improve high-quality economic development in China? The mediating effects of digital economy. Sustainability. 2022;14: 12143. doi:10.3390/su141912143

Spatial effects of digital economy and foreign direct investment—A U-shaped relationship. infse. 2023;4. doi:10.23977/infse.2023.040205

*8.* *Thus, this thesis aims to investigate the impact of the digital economy on green total factor productivity. The thesis word is not appropriate here.*

**Response**

We appreciate the reviewer#2’s careful comments. We have followed the reviewer’s suggestion by replacing the word “thesis” with the word “paper”. Therefore, the revised sentence is “Thus, this paper aims to investigate the impact of the digital economy on green total factor productivity.” (Line 142-145 of the revised manuscript)

*9. What is the DEA-SBM model?*

**Response**

We thank the reviewer for your professional comments. The explanation is as follows “The full name of DEA-SBM is data envelopment analysis - slack based measure. The DEA-SBM model is a method used in the field of operations research and production management to assess the efficiency of decision-making units. DEA is a non-parametric linear programming technique that evaluates the efficiency of DMUs by comparing them against a frontier formed by the best-performing units. The DEA-SBM model addresses this issue by incorporating these slacks directly into the efficiency score calculation. It provides a more comprehensive measure of efficiency by not only considering the radial distance but also the slacks in inputs and outputs.” (Line 84-90 of the revised manuscript)

*10. The introduction of the should have motivation, key objectives and contribution in a clear way.*

**Response**

We are very grateful to the reviewer#2 for careful review. We have followed the reviewer#2’s suggestion by revising the introduction extensively. The content is as follows“Despite prior studies conducted to reveal the impact of digital economy on green total factor productivity, there remains room for improvement in terms of indicator measurement and theoretical frameworks. Firstly, environmental pollution and resource consumption have not been included as part of the total factor productivity indicator, although China's total factor productivity have been measured in some studies [6, 7]. Therefore, traditional indicators have been modified and upgraded to measure China's green total factor productivity more accurately. Secondly, the "clean" attribute of digital economy plays a crucial role in driving the growth of green economy. However, the focus of previous research is placed on the improvement of economic efficiency [8], with the environmental perspective of the digital economy ignored. Therefore, this study aims to find out the relationship between digital economy and green total factor productivity. To achieve this purpose, the panel data from 2004 to 2019 for 30 provinces in China is used to measure their green total factor productivity respectively. Besides, Generalized Method of Moments model and threshold model are applied to empirically examine the effect of digital economy on green total factor productivity, along with the threshold effects of innovation environment and institutional environment.

The potential theoretical contributions of this study are detailed as follows. First, the DEA-SBM model is used to include relevant undesirable outputs in traditional total factor productivity, and the Malmquist-Luenberger productivity index is used to classify green total factor productivity by region. With new data perspectives provided, this contributes to the existing literature. Second, the existing literature on the green effects of digital economy is expanded from a macro perspective by exploring the impact of the digital economy on green total factor productivity. Finally, innovation environment and institutional environment are introduced as threshold variables to explore their influence exerted on the relationship between the digital economy and green total factor productivity. This is beneficial in deepening our understanding as to the green economic consequences of digital economy from the perspectives of institutional environment and innovation environment. ”

**Literature review and study hypotheses**

*11. The literature review is not synchronized. It looks like the summary of different studies. So, it should be improved by mentioning what has been done on this topic and explaining the literature gap that will be covered in this study.*

**Response**

We are very grateful to the reviewer#2 for careful review. We have followed the reviewer#2’s suggestion by revising literature review extensively. The content is as follows“ As an emerging economy, the digital economy is having a significant impact on the way the economy is developed today. The digital economy comprises a series of economic activities that rely on digital knowledge and information as key production factors, with modern information networks serving as important carriers. Effective utilization of information and communication technology plays a critical role in driving efficiency improvements and optimizing economic structure. In contrast to traditional economies, the digital economy pervades every aspect of social production. While existing research on the digital economy has primarily centered on its economic consequences, more recent efforts have focused more directly on two critical aspects of digital economy research. The first critical aspect centers on a shift away from direct analysis of the digital economy's economic consequences to a more focused study of intermediate mechanisms. Examples of such mechanisms include the relationship between the digital economy and technological innovation, financial infrastructure, environmental regulations, foreign direct investment, and more [9, 12]. The second crucial aspect involves a greater focus on exploring the interaction between the digital economy and other academic disciplines. For instance, [13] proposed that the digital economy can play a vital role in mitigating cyber threats and enhancing public welfare. Meanwhile, [14] investigated the impact of the digital economy on educational development and identified that it can significantly improve the quality of education. Notably, these studies have primarily focused on analyzing the direct or indirect impact of the digital economy on various production activities, with comparatively less attention devoted to exploring the impact of the digital economy in different contextual settings. To address this gap, the concept of green total factor productivity has emerged as an essential indicator for evaluating the quality of economic development. Green total factor productivity builds upon traditional total factor productivity accounting methodologies, incorporating factors such as resource consumption and pollution emissions to more fully capture unexpected outputs [15]. By integrating economic benefits with ecological resource protection, Green total factor productivity provides a more comprehensive evaluation of economic development. Research on green total factor productivity first involved the field of ecological and environmental. The current body of research on Green total factor productivity has predominantly explored its measurement and influencing factors. On the measurement of green total factor productivity, [16] started by introducing the DEA method into the calculation of total factor productivity. As the first to propose the directional distance function, [17] adopted it to calculate the Malmquist-Luenberger productivity index, which involves the emission of pollutants and other undesirable outputs in the total factor productivity index system. [18] developed the distance function in the non-radial SBM direction and proposed to calculate green total factor productivity by using the Luenberger index. This research has identified key factors that impact green total factor productivity, including economic factors such as capital investment and labor productivity [19], industrial structure [20], government regulations related to energy efficiency and pollution reduction [21], and environmental regulations aimed at reducing pollution and promoting sustainability [22]. For instance, [23] have suggested that China's outward direct investment can promote the green economy at the provincial level. [24] have investigated the interplay between government and environmental decentralization and have concluded that the environment's effectiveness in promoting regional green growth is diminishing over time. However, these studies have only considered individual factors that affect green total factor productivity. There are few systematic evaluations of the digital economy's impact on green total factor productivity. ”(Line 98-143 of the revised manuscript)

*12. Economic theories should support the hypothesis of the study.*

**Response**

We are very grateful to the reviewer#2 for careful review. We have followed the reviewer#2’s suggestion by adding economic theories to support the hypothesis of the study. The content is as follows:

According to the theory of technological progress, technological development plays a pivotal role in the improvement of productivity [25]. Under this context, digital technology contributes significantly to the development of digital economy. When popularized, it has the potential to enhance the efficiency of resource utilization, reduce production costs, alleviate environmental pressures, and ultimately improve the level of green total factor productivity. The theory of green growth attaches much importance to sustainable development and environmental preservation [26]. Green total factor productivity is considered as a critical indicator needed to evaluate environmental performance. In this regard, digital economy provides a large amount of data and information requisite for efficient environmental management and policy-making. To sum up, digital economy plays a pivotal role in shaping a sustainable future as it allows environmental challenges to be better understood and addressed, reduces the dependence on natural resources, mitigates pollution and carbon emissions, and enhance green total factor productivity. (Line 147-158 of the revised manuscript)

The Schumpeterian innovation theory emphasizes the critical role played by the innovation environment in shaping and influencing innovation in various aspects, including its types, quantity, and effects [38]. A favorable innovation environment is conducive to the development of digital technology and the upgrading of digital infrastructure, both of which are essential for growing the digital economy. (Line 205-209 of the revised manuscript)

**Research design**

*13. The research design of the study requires further explanation.*

**Response**

We are very grateful to the reviewer for careful review. We have followed the suggestion. We have refined our research design to enhance its clarity and comprehensibility. Specifically, we have employed generalized method of moments to investigate the relationship between the digital economy and green total factor productivity. Subsequently, a threshold model has been constructed, wherein innovation environment and institutional environment serve as threshold variables to explore the differential impact of the digital economy on green total factor productivity. (Line 247-274 of the revised manuscript)

**Data source and sample selection**

*14. It is important to provide the data source link of each variable in the study.*

**Response**

We have followed the reviewer’s suggestion by adding the data source link of each variable to the study. Correspondingly, we have added some explanations about the data source of variables in revised manuscript (Line 280-293 of the revised manuscript), as below: “The development indexes of digital economy are selected from the Chinese Research Data Services Platform which provides economic information services in China. The database can be searched on cnrds.com. Green total factor productivity data are selected from the China Statistical Yearbook and the China Statistical Yearbook on Environment. China Statistical Yearbook comprehensively reflects the statistical data of economic and social development of various provinces and regions in China. China Statistical Yearbook on Environment predominantly centers on environmental statistical data and pertinent information within China. It encompasses statistical data pertaining to environmental conservation, resource utilization, pollution control, and ecological conditions. Financial data are collected from the China Stock Market and Accounting Research Database. China Stock Market and Accounting Research Database is an important financial information database in China, associated with Guo-Tai-An firm. Primarily, the database collects, organizes, and disseminates various financial and economic data to facilitate risk assessment, market analysis, policy formulation, and decision-making for financial institutions, investors, researchers.”

*15. The green total factor productivity variable methodology has so many details. The authors explain how they combine the capital, energy, and labor inputs.*

**Response**

We have followed the reviewer’s suggestion by adding some explanations about the inputs of the capital, energy, and labor in revised manuscript (Line 347-365 of the revised manuscript), as below: “Green Total Factor Productivity is an indicator that measures production efficiency by considering both the resources required for production and the environmental impact of output. The primary resource inputs considered in Green Total Factor Productivity are capital, energy, and labor. The selection of capital, energy, and labor is driven by several reasons. First, these elements constitute the core components of the production process. Capital encompasses equipment, machinery, and other investments; energy stands as an indispensable resource in production activities, while labor represents human workforce. Evaluating these elements allows for a more comprehensive assessment of production efficiency and resource utilization within the production process. Second, the inclusion of energy as a factor is due to its close association with environmental impact. Assessing energy inputs aids in measuring the production's environmental footprint. Considering energy use allows for a better understanding of whether production is sufficiently efficient, thereby reducing resource wastage and environmental pollution. Third, capital and labor are comparable resource elements. This comparability facilitates easier comparisons of production efficiency among different industries, countries, or regions. With these elements sharing common units of measurement, it aids in analysis and research. Therefore, utilizing capital, energy, and labor as constituents of Green Total Factor Productivity contributes to a comprehensive evaluation of production efficiency while integrating environmental factors, thereby better supporting sustainable and environmentally friendly economic growth.”

*16. What’s the logic and reasoning for selecting the mentioned variables for the digital economy?*

**Response**

We appreciate the reviewer’s insightful comments. The reviewers' comments indicate that our exposition needs further fleshing out. We adopt the professional comments of the reviewer and provide the following explanation. Based on the meaning of digital economy, the Internet is an important carrier for the development of digital economy. The construction of digital economy indicators at the inter-provincial level takes Internet informatization as the core of the measurement. For example, Liu(2020) published an article on constructing the digital economy index through informatization and digitization indicators. For the selection of digital economy indicators, in line with the literature (Pan et al., 2020), our indicators include both total telecommunication services per capita, the share of the number of people in information computer services, and other information technology indicators. Informationization indicators reflect the current degree of informationization in the region and reflect the basic support environment of the development of the digital economy. Our indicators also include the number of Internet broadband access users, the number of cell phone users and other Internet development indicators, which fully consider the infrastructure of the digital economy and reflect the conditions for the development of the digital economy.

*17. The Fan Gang market index measures the institutional environment, so it is important to give the details and link to this index.*

**Response**

We appreciate the reviewer’s insightful comments. We have followed the reviewer’s suggestion by adding the details and link of the index to revised manuscript (Line 406-417 of the revised manuscript), as below: “The institutional environment constitutes an external factor that influences corporate behavior and decision-making. A favorable institutional environment implies equitable and transparent market competition. The degree of institutional environment improvement depends on the level of regional marketization. The Marketization Index of China’s Provinces, proposed by Fan Gang, is a metric for assessing the level of marketization in various regions of China. The comprehensive index takes into account several factors, including government-market relationships, non-state economic development, product market development, market service scale, and legal environment. The Index serves as a tool for governmental bodies, research institutions, and economists to gain insights into the marketization status of different regions in China, facilitating the formulation of policies aimed at promoting economic efficiency and growth. The Index, proposed by Fan Gang, can be accessed on cmi.ssap.com.cn.”

*18. How authors choose the control variables. What is the logic behind this? Are these the only variables that affect the TFP?*

**Response**

We appreciate the reviewer’s insightful comments. We choose control variables for the following reasons: First, it is imperative to explicitly define the research inquiry concerning the relationship between the digital economy and green total factor productivity, identifying the theoretical factors associated with both the digital economy and green total factor productivity. These factors are commonly perceived as potential control variables. Consequently, our choice of control variables is grounded in the theoretical underpinnings of the research. Second, a review of the literature reveals the control variables utilized by other researchers in exploring the nexus between the digital economy and green total factor productivity. References can be used as the basis for selecting control variables. Therefore, we choose the control variables according to the references. The references are as follows:

Han L, Chen S, Liang LL. Digital economy, innovation environment and urban innovation capability. Science Research Management. 2021; 42: 35–45.

Wu FF, Zhang XF. A study on the impact of local institutional environment on foreign exports of heterogeneous firms. Industrial Economics Research. 2018: 40–51.

Liu YS, Tian YH, Luo Y. Industrial structure upgrading, energy efficiency and green total factor productivity. The Theory and Practice of Finance and Economics. 2018; 39: 118–126.

Third, there may exist endogeneity issues between the digital economy and green total factor productivity. Control variables are instrumental in mitigating bias stemming from endogeneity. To address this, we have selected Control Variables (Human capital, Traffic condition, Industry structure, Research and development investment, The level of economic development, Foreign direct investment, The level of science and technology, The level of opening up ) to alleviate endogeneity concerns.

These variables are not the sole determinants affecting green total factor productivity. We posit the existence of latent variables influencing green total factor productivity. However, in this study, we opted for a Generalized Method of Moments to mitigate the impact of latent variables. Generalized Method of Moments allows for the consideration of the lagged effects of green total factor productivity, enabling a more comprehensive capture of latent variables that might manifest effects across multiple time periods. By introducing the lagged term of green total factor productivity as a control variable, Generalized Method of Moments provides a more holistic assessment of the influence of latent variables. (Line 443-464 of the revised manuscript)

**Empirical analysis**

*19. What’s the motivation to choose the fixed effect and GMM method?*

**Response**

We appreciate the reviewer’s professional comments. We choose the fixed effect and GMM method for the following reasons. We have followed the reviewer’s suggestion by adding the reasons to revised manuscript (Line 501-510 of the revised manuscript), as below: “To test the appropriateness of the GMM method setting, this paper conducts regression analyses on the lagged data of digital economy and green total factor productivity across 30 provinces in China using ordinary least square, fixed effect, and GMM method. The outcomes of the regression are presented in Table 5. The results indicate that the coefficients of L.gtfp are significant, suggesting an impactful relationship from the previous period's green total factor productivity on the current period. The L.gtfp coefficient of ordinary least square and fixed effect are 0.1631 and 0.1146, respectively. The L.gtfp coefficient of GMM method is 0.1558, which lies between the ordinary least square and fixed effect. It shows that the model fitting effect is good. In this paper, we choose GMM method for estimation.”

*20. Which IVs are used while applying the GMM method, and how are they suitable?*

**Response**

We appreciate the reviewer’s insightful comments.We have chosen our instrumental variables for the following reasons: First, instrumental variables should be related to the endogenous explanatory variables but should not be associated with the error term. Second, instrumental variables should not be correlated with the dependent variable; otherwise, they would lose their effectiveness in addressing endogeneity. We introduce the lagged one-period Green Total Factor Productivity as our explanatory variable, constituting a dynamic panel. We employ the lagged one-period Digital Economy as our instrumental variable for GMM method. To enhance the effectiveness of GMM method and address potential endogeneity issues in control variables, we include lagged terms of control variables such as road, indu, edu, pgdp, open, tech, for

in our instrumental variable set. To further validate the results of our GMM method, we conduct AR test and Hansen test.

*21. The results and discussion are weak. It is important to add the economic justification of the outcome and compare them with previous studies.*

**Response**

We appreciate the reviewer’s insightful comments. We have followed the reviewer’s suggestion by adding the discussion and the results to revised manuscript (Line 660-713 of the revised manuscript), as below:As shown in Table 6, the digital economy can significantly enhance green total factor productivity, which is consistent with the results in the literature [54, 55]. This paper used Generalized Method of Moments method to prove that digital economy can promote the green total factor productivity. Not only does the digital economy boost economic efficiency, but it also induces a positive impact on environmental protection [56]. The digital economy plays an essential role in the enhancement of green total factor productivity for two primary reasons. First, the development of the digital economy serves as a drive for technological innovation and progress, such as big data, internet of things, and artificial intelligence are applied to traditional industries in various provinces and stimulate green output growth [57]. Second, as a new type of production factor, the digital economy can integrate with input factors such as capital and energy in green total factor productivity, lower energy consumption, enhance energy utilization efficiency, transform the traditional extensive development mode, and explore new paths for green growth [58]. We found that the digital economy holds significant value in terms of green economics, which not only aligns with the principles of sustainable development but also boosts high-quality growth.

The impact of the digital economy on green total factor productivity exhibits a singular threshold effect in the innovation environment, which is consistent with the findings from previous studies. [59, 60] pointed that the development of the digital economy and the enhancement of green total factor productivity requires support from an innovation environment. In a sluggish innovation environment, provinces may be exposed to uncertainty in the process of economic growth. Inadequate impetus for digital technology gives rise to a tendency to allocate more important resources to high-profit production in order to reduce uncertainty in the external innovation environment. As a result, regions may not take advantage of the digital economy to improve green total factor productivity, and the digital economy exerts a significantly negative impact on green total factor productivity in this regard. Once the innovation environment exceeds a certain standard, digital economy advanced regions are more likely to invest in digital infrastructure and technological transformation [61]. With increased digitization, provinces will make use of the technological and innovative advantages of their own digital economy to phase out outdated production capacity, reduce pollution emissions, and enhance capacity utilization efficiency to boost the growth of green total factor productivity [62]. As shown in Table 7, the innovation environment has a nonlinear effect on the digital economy and the improvement of green total factor productivity, which indicates a threshold effect.

As suggested by [63, 64], there is a positive and linear relationship between the institutional environment and the promotion of the digital economy and green total factor productivity. A well-functioning institutional environment implies a fair market environment and the rational distribution of green resources, which can help enhance green total factor productivity [65]. However, and contrary to our expectation, we found that there is significant difference in the digital economy and green total factor productivity between regions with a low degree, a medium and a high degree of institutional environment. The impact of the digital economy on green total factor productivity exhibits non-linear characteristics. Besides, in regions with medium to low degree of the institutional environment, the improvement of the institutional environment not only contributes to the full play of institutional advantages but also stimulates the vitality of market economic entities in various regions, increases investment in digital technology research and development, drive digital reform, optimize internal resource allocation, and exert a positive influence on green total factor productivity of the digital economy, which demonstrates an increasing marginal returns of the digital economy. The marginal return of the digital economy in regions with a high level of institutional environment is lower than in regions with a medium-level institutional environment for the reason that the former boasts a more sophisticated legal foundation and market environment for the operation of the digital economy.

Given the relative scarcity of resources, they exhibit less tendency to allocate a large amount of vital resources to the development of the digital economy, which causes a diminishing marginal return as the level of digital economy development reaches a critical scale. In summary, the impact of the digital economy on green total factor productivity displays a dual threshold effect in the institutional environment.

*22. The quantile regression graph should be added for clear understanding.*

**Response**

We draw the quantile regression graph and add it to the revised manuscript. We explained the results of the quantile regression. The figure is as follows:

Fig.3 The quantile regression graph of the influence of digital economy on green total factor productivity

**Conclusions and research recommendations**

*23. The authors fail to conclude the study. The policy implication should be based on the findings.* *The limitations and future research directions are missing.*

**Response**

We have followed the reviewer’s suggestion by adding the limitations and future research directions to revised manuscript (Line 715-778 of the revised manuscript), as below: The digital economy is an important factor in promoting the efficiency of the green economy. The digital economy plays an important role in promoting green total factor productivity. The panel data of 30 provinces in China are used in this study during the period of 2004–2019. This paper analyzes the impact of the digital economy level on green total factor productivity by using the system Generalized Method of Moments and the panel threshold model. The paper draws the following conclusions and the policy implications.

First, the digital economy has a significant positive impact on the improvement of green total factor productivity, and has become a new driving force for green economic growth. This conclusion is still true after excluding special provincial samples, replacing models, and shortening sample time. Therefore, the digital economy should be vigorously developed in various provinces, basic industries of the digital economy such as big data and blockchain should be cultivated, accelerating the development of the digital economy industry, improving the construction of digital infrastructure, and digital industries should be integrated with traditional industries such as high energy consumption and high pollution. Moreover, digital technology should be applied to the field of ecological environmental protection to reduce pollutant emissions and improve overall green total factor productivity.

Second, the impact of the digital economy on China's green total factor productivity has a single threshold effect with the innovation environment as the threshold variable. The impact of the digital economy on green total factor productivity has changed from negative to positive. It shows that regions with a higher level of innovation environment are more motivated to carry out digital technology innovation and digital infrastructure construction, and gain the advantages of green innovation brought by the digital economy. Therefore, the investment of scientific and technological innovation in the field of environmental protection should be strengthened, intensifying green technology research and development, developing innovative digital economy industries vigorously, promoting the development of "industry-university-research". Furthermore, the integration of digital economy and talent strategy, foreign investment, technological upgrading, etc. should be strengthened, improving the innovation environment, integrating digital innovation into traditional industries, so as to improve green total factor productivity and realize green economic development.

Third, the impact of the digital economy on China's green total factor productivity has a double threshold effect with the institutional environment as the threshold variable. The effect of the digital economy on Gtotal factor productivity varies with the level of the institutional environment. And the positive effect is most significant when the institutional environment is between a certain interval value. When this value is crossed, the impact of the digital economy on green total factor productivity is weakened. This shows that the digital economy presents a nonlinear characteristic of China's green total factor productivity from marginal increase to marginal decrease. Therefore, it is suggested to strengthen the government's guiding role in the market. On the premise of better playing the role of the government, it is necessary to stimulate the vitality and dynamism of enterprises and the market, improve the institutional environment as well as governance, and formulate effective policies to improve the development of the digital economy. Additionally, the development of innovative digital industries should be greatly supported, actively cultivating investment entities in a diversified manner. Furthermore, it is essential to increase investment in the digital economy, so that it can fully integrate with all walks of life based on the high permeability of the digital economy, effectively promoting resource utilization and efficiency. At the same time, allocation efficiency should be improved and pollutant emissions should be reduced, improving the ecological environment, and promoting the digital economy continuously to promote green economic development.

Fourth, further analysis finds that the impact of the digital economy on China's green total factor productivity has different quantile effects. The regression conclusions are generally consistent with the previous empirical evidence. Therefore, the development of the digital economy should be rationally planned in various regions, strengthening the construction of digital soft and hard environments, improving digital infrastructure such as 5G and big data, and narrowing the gap in the development of the regional digital economy. Moreover, the development of the digital economy should be steadily promoted according to local conditions and scientific guidance, so as to fully release the dividends of the development of the digital economy, and contribute to green and high-quality economic development.

There are several limitations to this paper. First, its broader connotations were not fully considered for the construction of digital economic indicators due to the absence of a unified definition of the digital economy, such as industrial digitization and digital industrialization. Consequently, the measurement system used in this study is unable to reflect the level of digital economic development comprehensively and accurately. Besides, there are inherent weaknesses in the construction of the indicator system. For future research, it is recommended to explore digital economy at multiple levels and examine their impact on green total factor productivity. Second, there are many factors and mechanisms that can improve green total factor productivity, which requires further investigation. Future studies should be conducted to investigate the interaction between internal and external factors, which can improve the understanding of green total factor productivity. Third, although the dynamic effects of digital economy on green total factor productivity are identified in this paper, the spatial spillover effects associated with digital economy are still not addressed. Future research can focus on exploring how to improve the methods of modelling and testing.

*24. The language quality of the manuscript should be improved.*

**Response**

We apologize for the poor language of our manuscript. We have improved the English in the secondly revised manuscript. First, we polished the language by the editing service to improve readability of the manuscript. Second, we have double-checked the English to polish the language throughout the manuscript. Third, we have carefully corrected the typos found in our manuscript. We really hope that the flow and language level have been substantially improved.

*25. There should be symmetry in the whole manuscript.*

**Response**

We appreciate the reviewer#2’s careful comments. We have recognized the paramount importance of symmetry in the manuscript to ensure a well-organized and professionally crafted document. In accordance with the suggestions of the reviewer, the following adjustments have been made: First, in terms of formatting, we have ensured uniformity in the manuscript's formatting. This encompasses consistency in font style, size, line spacing, heading styles, and page layout. Second, in structuring the paper, we have optimized the flow and coherence of the content, ensuring seamless transitions and cross-references between sections. Third, we have conducted a comprehensive review to confirm the manuscript's overall consistency. Upon completing the manuscript, we conducted a final review to ensure uniformity in formatting, alignment, and the overall visual presentation.
